# Monitoring complete ammonia oxidizers bacteria: relevant players for nitrogen removal from wastewater

**DOI:** 10.1186/s13568-025-01878-6

**Published:** 2025-07-04

**Authors:** Javier Duque, Leire Besga-Oyanarte, Miguel De Celis, Susana Serrano, José Luis Alonso, Antonio Santos, Lucía Arregui

**Affiliations:** 1 Unit of Microbiology, Department of Genetics, Physiology and Microbiology, Complutense University of Madrid, Madrid, Spain; 2 Research Institute of Water and Environmental Engineering (IIAMA), Universitat Politècnica de València, Valencia, Spain

**Keywords:** Wastewater treatment plants, Nitrification, COMAMMOX bacteria, Fluorescent in situ hybridization (FISH), Digital PCR (dPCR), Next generation sequencing (NGS)

## Abstract

COMAMMOX (Complete Ammonia Oxidizers) bacteria seem to play an important role in nitrification, being able to directly transform ammonia into nitrates. Due to the relevance of this process in WWTPs, different approaches were used in this work to monitor COMAMMOX bacteria, as well as other microorganisms involved in the biological removal of nitrogen occurring in the bioreactor of a full-scale WWTP. This facility operates with low dissolved oxygen concentrations and prolonged sludge retention times. Fluorescence in situ hybridization confirmed the presence of COMAMMOX bacteria showing their distribution within the activated sludge flocs. Primers targeting the *amoA* gene, which encodes the A subunit of the ammonia monooxygenase enzyme, were employed as genetic markers in PCR techniques to simultaneously detect and amplify both COMAMMOX clades A and B, as well as specifically clade A, clade B, or various COMAMMOX *Nitrospira* species (Ca. *N. nitrosa*, Ca*. N. inopinata*, and Ca*. N. nitrificans*). Using digital PCR, a set of these primers enabled the estimation of the proportion of *amoA* COMAMMOX genes versus other nitrifiers (AOB, AOA and NOB). The data obtained not only confirmed the presence of COMAMMOX bacteria, but also their abundance, comparable to that of NOB and AOB. The 16S rRNA gene-based meta-taxonomy analysis gave complementary data about the microbial diversity and community composition within the samples. The results also highlight the biases of each technology used as monitoring tools, which must be accurate for the effective management of wastewater treatment plants (WWTPs) and for exploiting the potential of COMAMMOX bacteria.

**Supplementary Information:**

The online version contains supplementary material available at 10.1186/s13568-025-01878-6.

## Introduction

The pressing issue of water pollution underscores the urgent need to optimize sanitation systems. These systems rely on a series of infrastructures culminating in Wastewater Treatment Plants (WWTPs), which play a vital role in environmental protection. Among their primary objectives is the removal of nutrients, particularly nitrogen (N) and phosphorus. Nitrogen removal in WWTPs entails the transformation of ammonia (NH₄⁺) into nitrogen gas (N₂). While this transformation can be achieved chemically, biological nitrogen removal is both more environmentally sustainable and cost-effective. A comprehensive understanding of the microbial communities driving the nitrogen cycle is crucial for optimizing these processes and reducing operational costs (Holmes et al. 2019).

In urban WWTPs, nitrogen removal predominantly occurs through nitrification–denitrification pathways involving nitrate, with nitrification often being a critical step. Prior to the discovery of COMplete AMMonia Oxidizers (COMAMMOX) (Daims et al. 2015; van Kessel et al. 2015), nitrification was considered a two-step process requiring a division of labor between two distinct functional groups. In the first step, ammonia is oxidized to nitrite by Ammonia-Oxidizing Bacteria (AOB) and Ammonia-Oxidizing Archaea (AOA), utilizing ammonia monooxygenase (AMO) and hydroxylamine oxidase (HAO) enzymes. In the second step, nitrite is oxidized to nitrate by Nitrite-Oxidizing Bacteria (NOB) through the activity of nitrite oxidoreductase (NXR). The groundbreaking discovery of COMAMMOX bacteria revealed that a single microorganism could accomplish the complete oxidation of ammonium to nitrate via nitrite, a process that had previously been hypothesized (Costa et al. 2006). The first identified species were provisionally named Candidatus *Nitrospira inopinata* (Daims et al. 2015), Ca. *N. nitrosa*, and Ca. *N. nitrificans* (van Kessel et al. 2015). All known COMAMMOX bacteria belong to the genus *Nitrospira*, which was historically considered to consist exclusively of canonical nitrite-oxidizing species (Daims et al. 2015). However, these newly discovered species differ from their canonical counterparts by possessing a complete set of *amo* and *hao* genes within their genomes (van Kessel et al. 2015).

The genus *Nitrospira* is classified into six sublineages (Daims et al. 2015), which are widely distributed in natural environments and human-engineered systems (Daims, 2001; Daebeler et al. 2014; Feng et al. 2016). COMAMMOX bacteria identified to date belong to sublineage II, which also includes NOB lacking the enzymatic machinery required for reduced nitrogen compound oxidation. However, phylogenetic analyses based on 16S rRNA or *nxr* genes do not place COMAMMOX bacteria in a monophyletic clade within sublineage II due to the high sequence similarity between COMAMMOX and NOB. Differentiation between these groups requires additional genetic markers, such as the genes encoding enzymes responsible for aerobic ammonia oxidation—ammonia monooxygenase (AMO) and hydroxylamine dehydrogenase (HAO) (Daims et al. 2015; van Kessel et al. 2015). Specifically, the *amoA* gene, encoding the A subunit of ammonia monooxygenase, is a widely used marker for identifying ammonia-oxidizing bacteria and archaea. Primers targeting the *amoA* gene have been developed to detect COMAMMOX bacteria using PCR techniques. These primers enable the simultaneous detection of clades A and B (Bartelme et al. 2017; Fowler et al. 2018; Wang et al. 2018; Zhao et al. 2019), as well as the specific detection of clade A (Pjevac et al. 2017; Xia et al. 2018; Fujitani et al. 2020; Jiang et al. 2020) or clade B (Pjevac et al. 2017; Jiang et al. 2020). Furthermore, primers have been designed to detect multiple *Nitrospira* species (Beach and Noguera 2019). Among clade A COMAMMOX bacteria, Candidatus *N. nitrosa* is the most commonly detected species in WWTPs, though Ca. *N. inopinata* and Ca. *N. nitrificans* have also been identified. Two of these species (Ca. *N. nitrosa* and Ca. *N. nitrificans*) were visualized using fluorescence microscopy with specifically designed rRNA-targeted probes via fluorescence in situ hybridization (FISH) (van Kessel et al. 2015).

Environmental and operational parameters, such as temperature, solids retention time (SRT), and nitrogen and oxygen concentrations, have been linked to the abundance of *Nitrospira* in WWTPs (Huang et al. 2010; Mehrani et al. 2020; Gruber et al. 2021). A microaerobic dissolved oxygen environment (below 0.2–1.0 mg/L), low ammonium concentrations, and extended SRTs appear to favor COMAMMOX bacteria over canonical nitrifiers (Roots et al. 2019; Cotto et al. 2020). These conditions were observed in a WWTP located in the Community of Madrid, Spain. This facility employs advanced infrastructure and treatment designs to produce high-quality treated water, which is discharged into nearby riverbeds to support ecological restoration. Additionally, the facility is housed within an industrial building to minimize its visual impact, while covered reactors minimize noise and odor emissions.

Developing efficient and accurate methods to detect, identify, and quantify nitrifying organisms (AOB, AOA, NOB, and COMAMMOX) remains a priority in COMAMMOX research, as the relative contributions of these populations are not yet fully understood (Xu et al. 2021; Latocheski et al. 2022). To address this, DNA-based methods—including FISH, conventional and digital PCR, and 16S rRNA gene sequencing—were employed to detect, quantify, and identify COMAMMOX bacteria in samples collected from the oxic compartment of the aforementioned WWTP’s bioreactor. The data obtained were validated and used to assess the accuracy of the applied methodologies.

## Material and methods

### WWTP description

The wastewater treatment plant (WWTP) serves 40,000 equivalent inhabitants and has a treatment capacity of up to 12,000 m^3^/day. Secondary treatment employs prolonged oxidation biological processes. The plant operates two water lines, each comprising an anoxic/oxic (A/O) reactor and a submerged membrane bioreactor (MBR) with ultrafiltration membranes. Influent enters directly into the anoxic zone, equipped with mixing agitators and hosting an external recirculation line for biological sludge from the membrane tanks. The oxic zone features an aeration system providing oxygen. The bioreactor has a surface area of 8388 m^2^, and 30% corresponds to the anoxic zone. Phosphorus removal is enhanced chemically to complement nitrogen removal. The MBR incorporates a hollow-fiber membrane ultrafiltration system downstream of the bioreactor. A vacuum pump draws treated water through these membranes, directing the permeate for disinfection or discharge.

### Sampling

Four mixed liquor samples were collected over three years: January (2020 and 2023), May (2021), and November (2021). Each 1 L sample was taken through a manhole located at the central part of the oxic zone of the biological reactor during turbine operation. This procedure ensured the representativeness of the samples, as the mixed liquor in the biological reactor was homogenized. The sample was transported under refrigeration and stored in the laboratory at 4ºC, where it was processed within the first 48 h. Facility managers provided influent, effluent, and mixed liquor characteristics, alongside operational parameters for the oxic bioreactor compartment. Analytical procedures adhered to the “Standard Methods for the Examination of Water and Wastewater” (APHA, 2017). Recorded values (Fig. S1) included treated flow, BOD₅ (Biological Oxygen Demand), TSS (Total Suspended Solids), COD (Chemical Oxygen Demand), N–NH_4_⁺ (Ammonia Nitrogen), N–NO_3_⁻ (Nitrate Nitrogen), TN (Total Nitrogen), TP (Total Phosphorous), and pH for influent and effluent, as well as MLSS (Mixed Liquor Suspended Solids), MLVSS (Mixed Liquor Volatile Suspended Solids), V30, pH, HRT (Hydraulic Retention Time), and SRT (Sludge Retention Time) for mixed liquor, and BOD_5_, TSS, COD, N–NH_4_ +, N–NO_3_−, TN, TP and pH from effluent.

Approaches for COMAMMOX monitoring.

Table 1 summarizes the four approaches used and their specific applications.

**Table 1:** COMAMMOX monitoring with various nucleic acid-based methods

Approach	Sample	Application	
		General	This study
Fluorescent in situ Hybridization (FISH)	January 2020	Microbial detection Microbial quantification	Detect, quantify and explore activated sludge floc location of AOB, NOB and COMAMMOX *Ca. N. nitrosa* and *Ca. N. nitrificans* species
Conventional PCR	May 2021, November 2021	Microbial detection	Nitrifying COMAMMOX *amoA* genes detection: both clades A and B simultaneously, specifically clade A or clade B or several COMAMMOX *Nitrospira* species (*Ca. Nitrospira nitrosa*, *Ca. Nitrospira inopinata and Ca. Nitrospira nitrificans*) in two seasonally different samples in which various DNA extraction procedures were applied. Other key genes detection: rRNA16S, *cynS* (NOB), *nxr* (*Nitrospira*) genes, and *amoA* gene of AOB and AOA
Digital PCR (dPCR)	January 2020, November 2021	Microbial quantification	Quantification of rRNA16S, *cynS* (NOB), *nxr* (*Nitrospira*) genes, and *amoA* gene of AOB in *Betaproteobacteria*, COMAMMOX *Nitrospira* clade A and B and AOA
NGS: sequencing of the 16S rRNA gene	January 2023, November 2021, May 2021, January 2020	Microbial characterization Microbial quantification	Activated sludge microbial community characterization. Determination of relative abundance of nitrogen related phylotypes and COMAMMOX *Ca. N. nitrosa* among samples

### Fluorescence in situ hybridization (FISH)

Subsamples (3 mL) were fixed with ethanol (v/v). Detection of Candidatus *N. nitrosa* and Ca*. N. nitrificans* utilized probes (Ntspa 476 and CNtspa476) developed by van Kessel et al. (2015). These and additional probes (Table S1) were 5′ labeled with FAM (EUB338 mix) or TAMRA dyes. Hybridization followed the protocol by Daims et al. (2004) at 46 °C. Post-hybridization, slides were air-dried and embedded in Vectashield (Vector Laboratories, USA). Samples were examined under an Olympus BX50 microscope equipped with specific filter sets and a 100 × oil immersion objective. Quantification involved capturing 20 image pairs per probe using green (FAM) and red (TAMRA) fluorescence filters. The images were analyzed using a custom program developed in MATLAB (Borrás, 2008), which calculates the area occupied by the fluorescence signal in pixels. The software quantifies the pixel area corresponding to the specific probe and compares it to that of the general bacterial probe (EUBMIX). The result is expressed as the percentage of the area hybridized with the specific probe relative to the total area detected by the general probe.

### DNA extraction protocols

To enhance COMAMMOX detection, DNA extraction was optimized, considering the presumed low abundance of COMAMMOX bacteria in the bioreactor and their likely aggregation within the floc. Three 250 mL aliquots were prepared: MC (control, untreated), MA (10 min agitation and 10 min sonication), and MB (20 min agitation and 15 min sonication). Each aliquot underwent alternative extraction protocols (Fig. S2), from which duplicate samples were subsequently processed using the DNeasy® UltraClean® Microbial Kit (QIAGEN) according to the manufacturer's instructions. DNA yield and quality were assessed using a NanoDrop™ 2000/2000c.

### Conventional PCR

Two primer sets were selected:

*Set 1* Targeted the *amoA* gene, which is common in all described COMAMMOX, and others specific to *Nitrospira* species (Ca. *N. nitrosa*, Ca. *N. inopinata*, and Ca*. N. nitrificans*) (Table S2). PCR conditions included 94 °C for 5 min, followed by 40 cycles of 94 °C for 30 s, 48 °C for 45 s, and 72 °C for 1 min, with a final elongation at 72 °C for 10 min.

*Set 2* Included generic primers for the 16S rRNA gene and specific primers for *amoA* genes of *Nitrospira* clades A and B (Table S2). Other primers were also used to detect different groups of nitrifying microorganisms. PCR conditions varied slightly, with a 95 °C initial denaturation for 10 min, followed by 40 cycles of 95 °C for 30 s, 55 °C for 30 s (or 45 s for *Nitrospira nxr* gene), and 72 °C for 30–45 s, concluding with a 10-min elongation at 72 °C.

All reactions used ReadyMix® Taq PCR Reaction Mix (Sigma-Aldrich), primers, and 150 ng of DNA, with water added to a 25 μL reaction volume. Products were purified with the GFX™ PCR DNA and Gel Band Purification Kit (GE Healthcare) and confirmed via electrophoresis on 1% agarose gels.

### Digital PCR (dPCR)

A digital polymerase chain reaction protocol for the analysis of activated sludge was developed. Reactions (40 μL) included 13.3 μL QIAcuity™ EG PCR Master Mix 3 × (Qiagen), primers (20 μM, Table S2), 5 μL DNA, and 19.7 μL water. Samples were processed in QIAcuity Nanoplate 26 k 24-well plates using the QIAcuity™ One thermocycler with software version 1.0.0.84, operated using the QIAcuity Software Suite version 1.2.18. Conditions included 95 °C for 10 min, followed by 40 cycles of 95 °C for 30 s, 55 °C for 30–45 s, and 72 °C for 30–45 s. After PCR, the amplification target is detected by measuring the fluorescence in all positive partitions. The QIAcuity Software Suite estimates the fraction of positive and negative reactions. As stated in the QIAcuity® Application Guide the fraction of positive reactions is then fitted to a Poison distribution to determine the absolute copy number of the target DNA molecule in the input reaction mixture in units of copies /μL and then automatically calculate the starting target DNA concentration in the form of copies /μL in the sample. Additional columns display the average values calculated from the replicates (www.qiagen.com).

### NGS: 16S rRNA gene sequencing

DNA concentrations were assessed using the Qubit 2.0 Fluorometer. Libraries were prepared at the Complutense University’s Genomics Unit following Illumina's "16S Metagenomic Sequencing Library Preparation" manual. The V3–V4 region of the 16S rRNA gene was amplified with 341F and 805R primers and Illumina adapters. Libraries underwent quality control with a Bioanalyzer 2100 (Agilent Technologies) and were pooled for sequencing on Illumina MiSeq (2 × 300 reads, MiSeq Reagent Kit v3). Sequence reads were processed using the DADA2 v1.26.0 package (Callahan et al. 2016) modeling and correcting Illumina-sequenced amplicon errors and generating Amplicon Sequence Variants (ASVs). We used the function filterAndTrim (truncLen = c(285, 195), maxN = 0, maxEE = 6, truncQ = 2, trimLeft = 20) from the DADA2 R package to filter and trim input fastq files. We obtained 1,397,309 good-quality reads, averaging 279,462 ± 145,956 per sample after filtering, aligning paired reads and chimera removal. The taxonomic assignment was performed using the naïve Bayesian classifier implemented in DADA2 using as reference the MiDAS 4.8.1 database (McIlroy et al. 2015). The Illumina sequencing raw data were deposited in the National Center for Biotechnology Information (NCBI) under accession number PRJNA1232652. Compositional analyses were performed in R and plotted with ggplot2 v3.5.1 R package (Wickham 2016).

## Results

### Detection and quantification of COMAMMOX by FISH

**Fig. 1:**
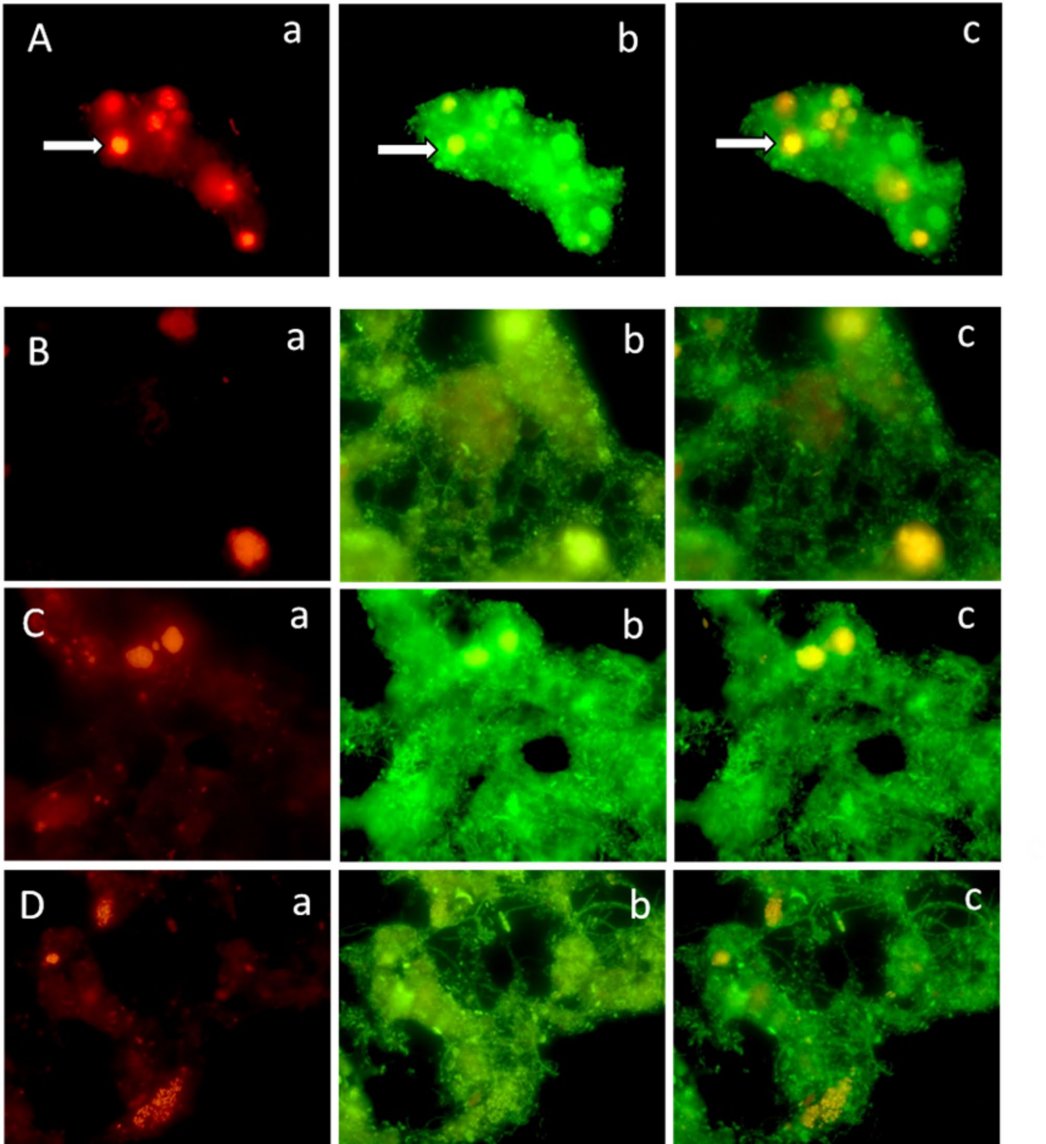
Microphotographs of samples taken from the bioreactor oxic chamber. **A** Selected field from the microscopical observations, 1000x: (a) COMAMMOX bacteria (*Ca. N. nitrosa, Ca. N. nitrificans*) detected with the Ntspa0476 probe (red emission filter), white arrow; (b) Communities belonging to the Eubacteria domain detected with the EUB mix 338I, 338II, 338III and 338IV probe and COMAMMOX bacteria (*Ca. N. nitrosa, Ca. N. nitrificans*) with the Ntspa0476 probe (green emission filter), white arrow, (c) Communities belonging to the Eubacteria domain detected with the EUB mix 338I, 338III and 333IV probe and COMAMMOX bacteria (*Ca. N. nitrosa, Ca. N. nitrificans*) with the Ntspa0476 probe (orange) (double emission filter), white arrow. **B**–**D** Random microscopical fields, 1000x. **B**: (a) COMAMMOX bacteria detected with Ntspa0476 probe (red emission filter) (b) EUB mix 338I, 338II y 338III and 338IV probe (green emission filter), (c) Ntspa0476 (orange) and EUB mix 338I, 338II y 338III and 338IV probes (double emission filter). **C**. *Nitrospira* detected with the Ntspa662 probe (red emission filter) (b) EUB mix 338I, 338II y 338III and 338IV probe (green emission filter), (c) Ntspa662 (orange) and EUB mix 338I, 338II y 338III and 338IV probes (double emission filter).** D** Betaproteobacteria AOB detected with the Nso1225 probe (red emission filter) (b) EUB mix 338I, 338II y 338III and 338IV probe (green emission filter), (c) Nso1225 (orange) and EUB mix 338I, 338II y 338III and 338IV probes (double emission filter)

The presence of COMAMMOX species was observed in activated sludge samples from the bioreactor using probes targeting the 16S rRNA gene of Ca*. Nitrospira nitrosa* and Ca*. Nitrospira nitrificans*. Dense, spherical-shaped cell aggregates were detected primarily on the surface of the flocs, with some also present in deeper zones (Fig. 1A, 1B). Nitrite-oxidizing bacteria (NOB) and ammonia-oxidizing bacteria (AOB) were also identified and quantified (Fig. 1C, 1D). Among the seven FISH probes employed, one detected NOBs and two targeted AOBs. The dominant genus responsible for nitrite oxidation was *Nitrospira* (Ntspa662 probe), with a relative abundance of approximately 3% (Fig. 1C). Neither *Nitrotoga* nor *Nitrobacter* genera were detected. The Nso1225 probe indicated a similar abundance for *Betaproteobacteria* AOBs, particularly within the *Nitrosomonadaceae* family (Fig. 1D).

Within the AOB community, halotolerant *Nitrosomonas* (NEU probe) exhibited a relative abundance close to 1%. Neither *Nitrosococcus mobilis* nor the *Nitrosospira* lineage were detected. The Eub338 probes were used to visualize total bacterial populations. Table 2 summarizes the nitrifying community composition determined by FISH.

**Table 2:** Results of the quantification of nitrifying species using the FISH technique

	Bacterial species/group	Abundance/uncertainty
COMAMMOX	*Ca. N. nitrosa, Ca. N. nitrificans*	3 ± 0.7%
NOB	*Nitrospira* spp.	3 ± 0.3%
	*Nitrobacter* spp.	0
	*Nitrotoga*	0
AOB	Betaproteobacteria ammonia-oxidizing bacteria	3 ± 0.8%
	*Nitrosococcus mobilis*	0
	*Nitrosospira* spp.	0
	Most halophilic and halotolerant *Nitrosomonas* spp.	1 ± 0.4%

### Detection of nitrifying COMAMMOX *amoA* and other key genes by conventional PCR

**Fig. 2:**
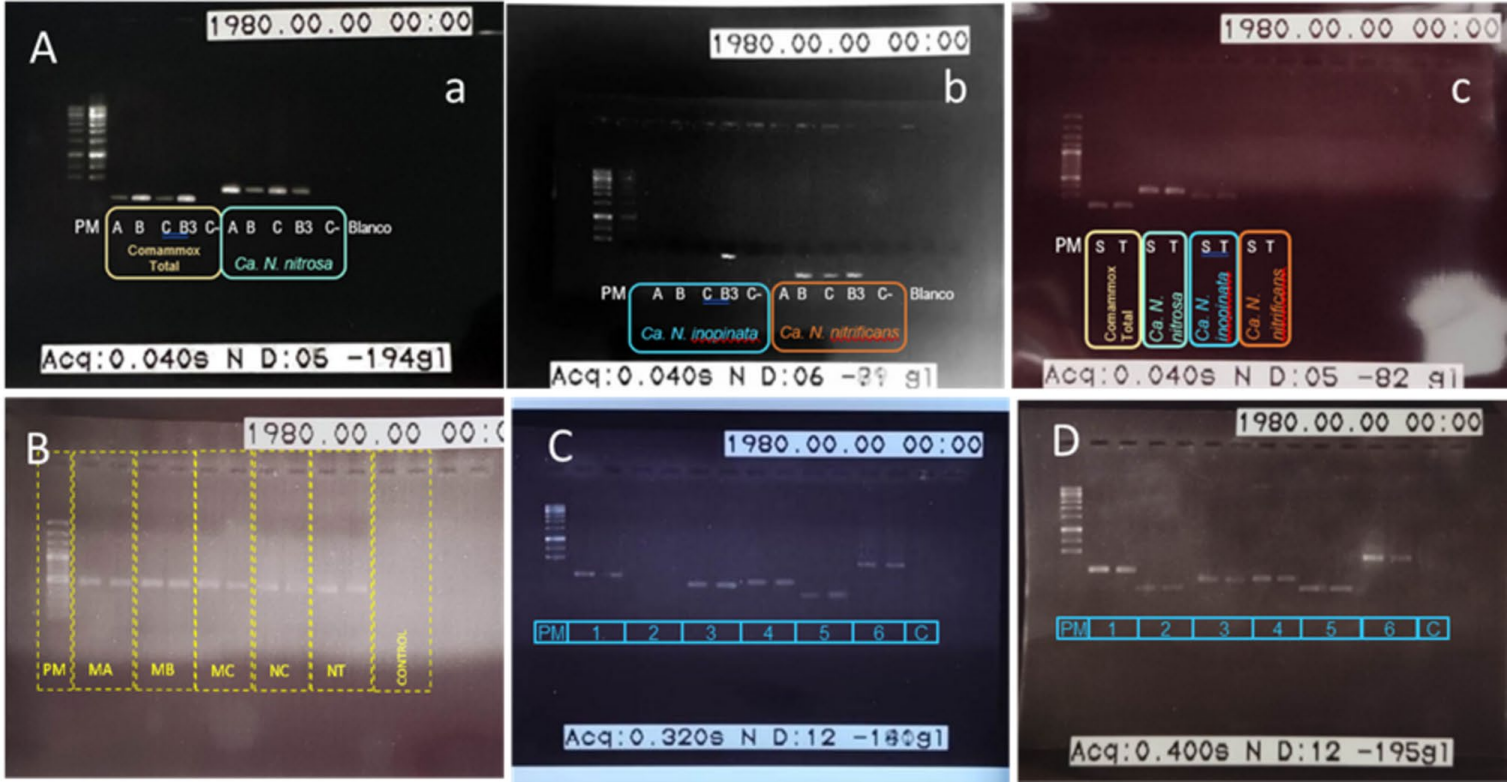
Electrophoresis gel assays carried out with the DNA extracted from samples subjected to different procedures. **A** (a,b). May sample. (c) November sample. Indicated COMAMMOX *amo A* gene amplification: (A) pre-treated sample, (B) pre-treated sample, (C) non-treated sample, (B3) sample obtained from the filter after filtration of a pre-treated sample, (C-) primer control, (S) non-treated sample (T) pre-treated sample. **B***Nitrospira nxr* gene amplification: (MA, MB) pre-treated May samples, (MC) non-treated May sample, (NC) non-treated November sample, (NT) pre-treated November sample. **C.** pre-treated May sample. **D** non-treated November sample. Amplification of the following genes: (1) COMAMMOX *Nitrospira* clade A *amo A* gene, (2) COMAMMOX *Nitrospira* clade B *amo A* gene, (3) NOB *cynS* gene, (4) AOA *amoA* gene, (5) Beta proteobacteria AOB *amo A* gene, (6) 16S rRNA gene. PM refers to the molecular weight standard

Amplicons of the expected length were generated using DNA extracts and published primers (Table S2). Total COMAMMOX bacteria were detected in both 2021 samples. In addition, Ca. *Nitrospira nitrosa* and Ca. *Nitrospira nitrificans* were identified in the May 2021 sample, along with Ca. *Nitrospira inopinata*, which was uniquely detected in the sample coming from the filter of one of the pre-treated samples (Fig. S2, Fig. 2Ab). In the November 2021 sample, Ca. *Nitrospira nitrosa* and faint bands of Ca. *Nitrospira inopinata* were observed on the electrophoresis gels (Fig. 2Ac).

Using the second primer set, amplification of the *amoA* gene for COMAMMOX *Nitrospira* clade A, the *cynS* gene for NOB, and the *amoA* genes of AOA and AOB were achieved. Additionally, the *nxr* gene from *Nitrospira* and the 16S rRNA gene of total bacteria were detected (Fig. 2B-D). The *amoA* gene of COMAMMOX *Nitrospira* clade B was observed only in the November 2021 sample, with a faint band on electrophoresis gels (Fig. 2D).

### Gene quantification by digital PCR (dPCR)

**Fig. 3:**
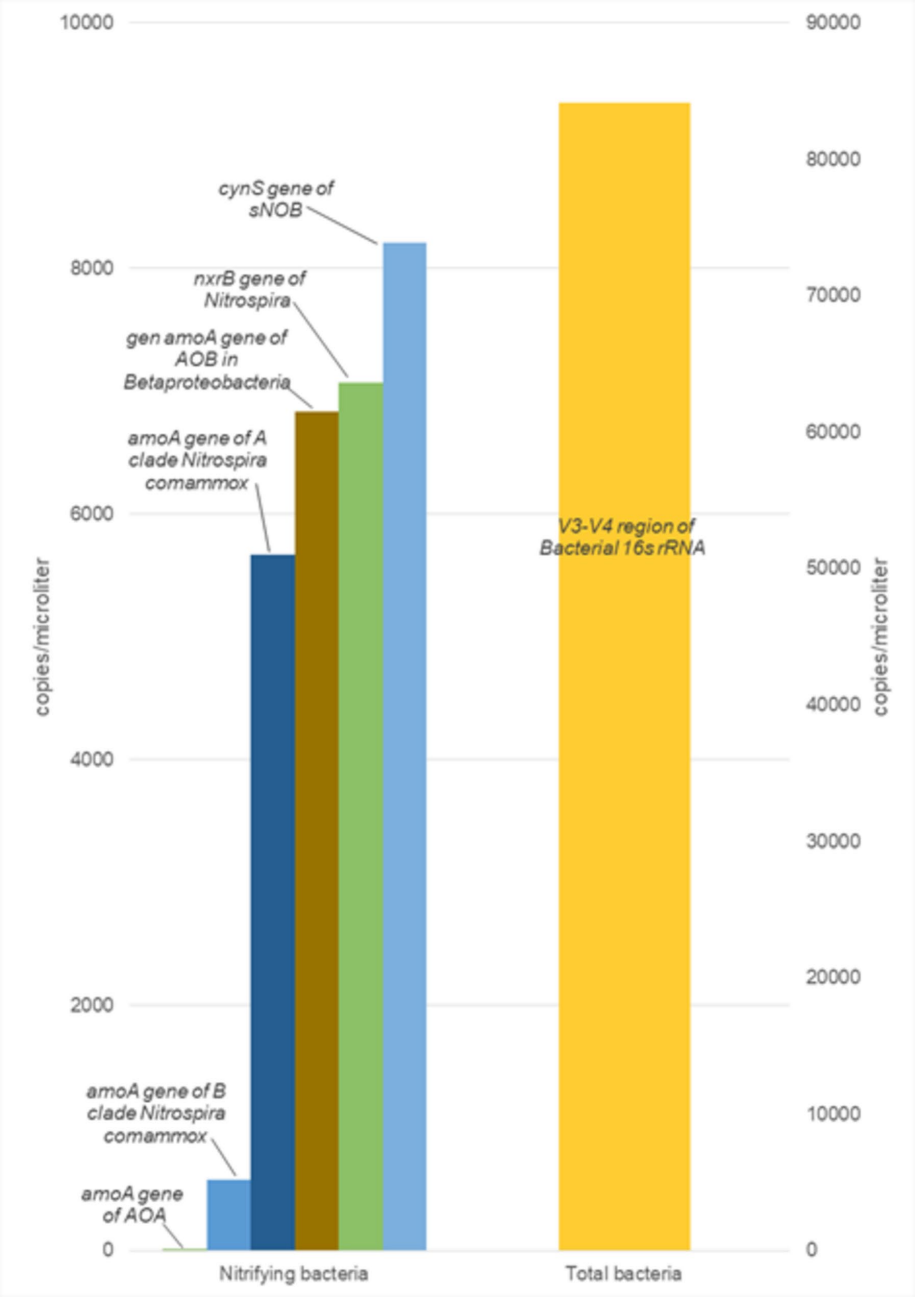
Digital PCR. Average concentration, in copies/μL, of each of the targets analyzed by dPCR corresponding to nitrifying bacteria/archaea within the samples. Primary y-axis refers to nitrifying bacteria and archaea and secondary y-axis refers to total bacteria

Quantification results are presented in Fig. 3 and detailed in Table 3. A total of 15,044.55 copies/μL (including *cynS* NOB and *amoA Betaproteobacteria* AOB) were detected for canonical nitrifiers. Regarding the detection and quantification of *Nitrospira*, it was observed that nearly all quantified strict NOB (*cynS* gene: 8213.20 copies/μL) likely belong to this genus (*nxrB* gene: 7065.80 copies/μL).

**Table 3:** DNA concentration (copies/μL), Confidence Interval (CI), Mean and Standard Deviation (SD) for the different targets analyzed by dPCR

Gene	Copies/µL	CI (95%)	Mean	SD	% Relative to 16S rRNA
AOA *amoA* gene	1.8	0.331	1.45	0.49	0.002
	1.1	0.427			
BOA *amoA* gene	6894.8	0.006	6831.35	89.73	8.1
	6767.9	0.006			
*Nitrospira* COMAMMOX clade A *amoA* gene	5448.8	0.006	5668.40	310.56	6.7
	5888.0	0.006			
*Nitrospira* COMAMMOX clade B *amoA* gene	737.9	0.017	580.30	222.88	0.7
	422.7	0.022			
sNOB *cynS* gene	8002.2	0.005	8213.20	298.40	9.8
	8424.2	0.006			
*Nitrospira NxrB* gene	6818.2	0.005	7065.80	350.16	8.4
	7313.4	0.005			
*16s rRNA Bacteria* gene V3–V4 region	82,960.1	0.005	84,140.40	167.30	100
	85,320.7	0.005			

Both COMAMMOX clades were present, with clade A predominating over clade B. Specifically, the concentration of clade A (5668.40 copies/μL) was one order of magnitude higher than that of clade B (580.30 copies/μL). Finally, it is noteworthy that this technique enabled the quantification of the gene encoding the *amoA* enzyme of ammonia oxidizing archaea, albeit resulting a very low value (1.45 copies/ μL).

### WWTP microbiome composition

#### Taxonomic profile of the bioreactor oxic compartment

**Fig. 4:**
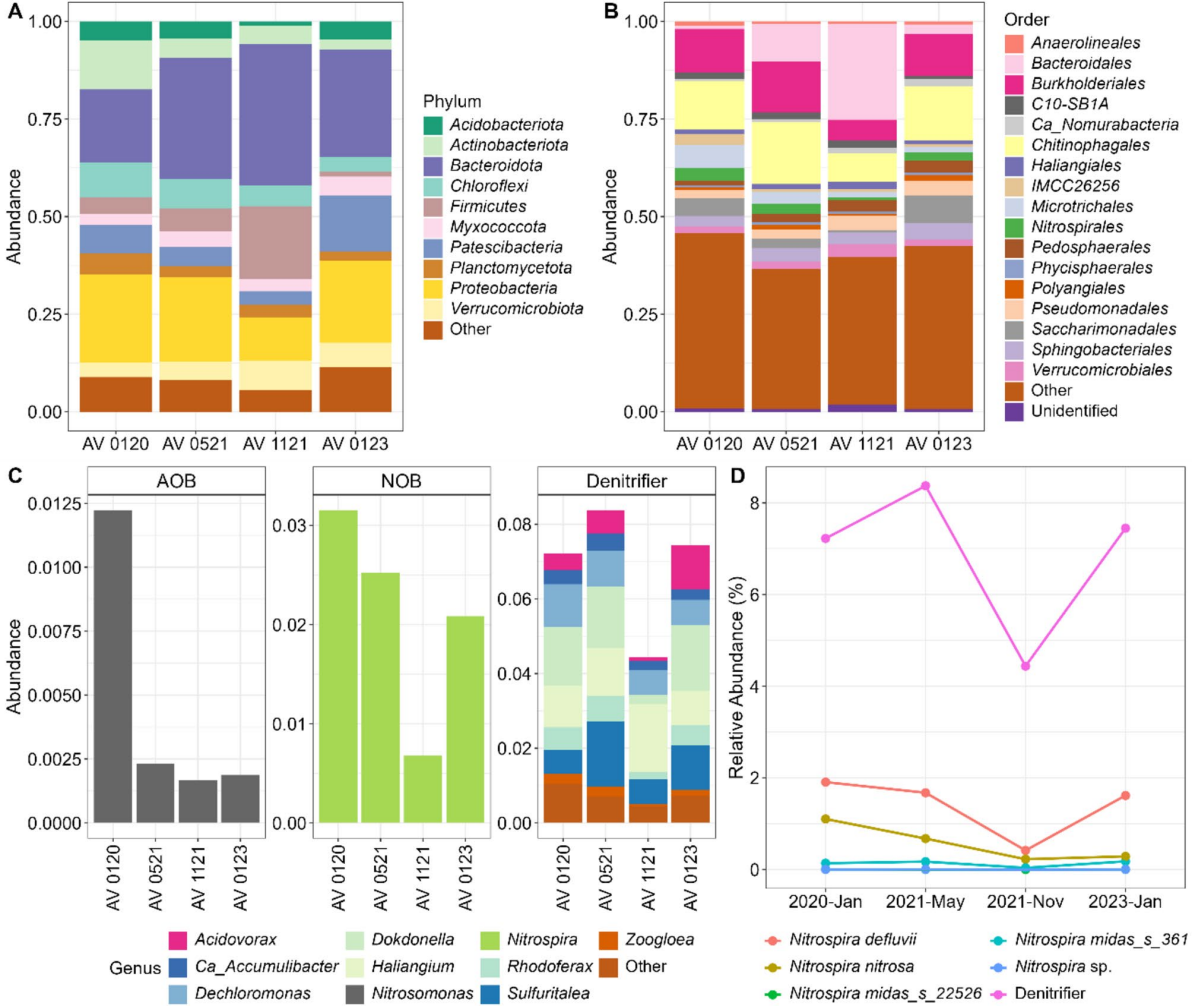
Taxonomic composition of WWTP samples. Relative abundance of dominant bacterial **A** phyla and **B** order. Phyla with less than 1% and orders with less than 0.5% of relative abundance are included in the “Other” category. **C** Relative abundance of nitrogen related phylotypes identified at the genus level. **D** Relative abundance (%) of *Nitrospira* species compared to the abundance of denitrifying bacteria

All samples show similar taxonomic profiles at the phylum level. The most abundant were the phyla *Bacteroidota* and *Proteobacteria*, (Fig. 4A), with notable presence also observed in *Acidobacteria*, *Actinobacteriota*, *Chloroflexi*, *Firmicutes*, *Myxococcota*, *Patescibacteria*, *Planctomycetota* and *Verrucomicobiota.* However, their relative abundance varied among samples. The most remarkable compositional change occurs in the November 2021 sample, with a significant increase in the relative abundance of the phyla *Bacteroidota* and *Firmicutes*, and a decrease in *Proteobacteria* (Fig. 4A). Within the phylum *Bacteroidota*, which is among the most abundant in these types of systems, the order *Bacteroidales* stands out, explaining the pattern of increased abundance in November, alongside a decrease mainly in the orders *Burkholderiales* and *Chitinophagales* (Fig. 4B).

### Nitrogen cycle-related populations

NOB populations consistently outnumbered AOBs in all samples. Only *Nitrospira* species were detected among NOBs, while AOBs were dominated by *Nitrosomonas*. NOBs were most abundant in January 2020 (3.15%), a value consistent with FISH results. On the other hand, AOBs constituted 1.22% in January 2020 (less than half of that indicated by FISH) but decreased to ~ 0.2% in subsequent samples. NOBs experienced the sharpest decline in November 2021.

Denitrifying bacteria were dominated by the genera *Acidovorax*, Ca. *Accumulibacter*, *Dechloromonas*, *Dokdonella*, *Haliangium*, *Rhodoferax*, *Sulfuritalea*, and *Zooglea* (Fig. 4C).

### *Nitrospira* genus and COMAMMOX species

Five *Nitrospira* species were identified, with *Nitrospira defluvii* being the most abundant nitrite oxidizer. *Nitrospira nitrosa*, a COMAMMOX species, was present in all samples, peaking in abundance in January 2020 and gradually declining in later analyses (Table 4, Fig. 4D).

**Table 4:** Relative abundance (%) of *Nitrospira* spp.

*Nitrospira* species	January 2020	May 2021	November 2021	January 2023
*Nitrospira* midas_s_22525	0.004	0.000	0.000	0.002
*Nitrospira* midas_s_361	0.137	0.172	0.037	0.178
*Nitrospira defluvii*	1.906	1.676	0.416	1.615
*Nitrospira nitrosa*	1.102	0.675	0.225	0.286
*Nitrospira* spp.	0.001	0.003	0.000	0.002

## Discussion

In this study, we present and compare the detection, identification, and quantification of COMAMMOX bacteria in the bioreactor of an advanced wastewater treatment plant (WWTP) in Madrid, Spain, using various molecular techniques. Since their discovery in 2015, COMAMMOX bacteria have garnered significant interest due to their potential advantages in nitrogen removal from wastewater, outperforming canonical nitrifying bacteria in certain conditions (Kits et al. 2017). Their ability to thrive under low dissolved oxygen levels (Xu et al. 2021) suggests that reducing aeration in biological reactors could lower energy consumption and operational costs in WWTPs (Luo et al. 2022). Additionally, COMAMMOX bacteria exhibit various metabolic benefits, including a lower contribution to N_2_O emissions, a key greenhouse gas (Zhu et al. 2022). While *N. inopinata* may produce N_2_O abiotically from hydroxylamine (NH_2_OH) (Kits et al. 2019), its emissions are significantly lower than those of ammonia-oxidizing bacteria (AOB). Promoting COMAMMOX processes could thus help mitigate nitrification-dependent N_2_O emissions and contribute to global warming reduction (Zhu et al. 2022). Furthermore, *N. inopinata*, the only COMAMMOX species currently isolated and cultured, has demonstrated the cometabolic degradation of certain emerging pollutants, including carbendazim, which remains untransformed by other ammonia-oxidizing microorganisms such as AOA and AOB (Han et al. 2019).

To confirm the presence and spatial distribution of COMAMMOX bacteria within the activated sludge floc, fluorescence in situ hybridization (FISH) was initially conducted on a sample from the WWTP bioreactor. The Ntspa 476 probe, originally developed by van Kessel et al. (2015) for detecting Ca. *N. nitrosa* and Ca. *N. nitrificans*, was later evaluated for specificity and coverage by Roots et al. (2019). Their findings indicated that the probe was insufficient for distinguishing COMAMMOX from canonical nitrite-oxidizing *Nitrospira* but effectively targeted a subset of lineage II *Nitrospira*, which includes known COMAMMOX strains. Microscopic examination of the sample revealed dense spherical clusters characteristic of nitrite-oxidizing bacteria (NOB) when hybridized with the Ntspa 476 probe. Similar bacterial aggregates were observed with the Ntspa662 probe, which specifically targets canonical *Nitrospira*, the sole NOB genus detected among the three tested (*Nitrobacter*, *Nitrospira*, and *Nitrotoga*). In contrast, AOB stained with the Nso1225 probe exhibited a more porous and non-spherical morphology, likely reflecting the different oxygen affinities among nitrifying groups (Law et al. 2019). While several genera of AOB exist (*Nitrosomonas*, *Nitrosospira*, *Nitrosolobus*, and *Nitrosovibrio*), phylogenetic analysis based on 16S rRNA does not definitively separate *Nitrosospira*, *Nitrosolobus*, or *Nitrosovibrio* at the genus level (Daims and Wagner 2010). FISH assays confirmed the presence of *Nitrosomonadaceae* members, aligning with prior studies identifying *Nitrosomonas* as the predominant AOB in WWTPs (Law et al. 2019). Notably, the relative abundance of AOB and COMAMMOX was similar, suggesting that their coexistence is driven by distinct ammonium affinities, allowing niche differentiation rather than direct competition (Cotto et al. 2020).

Due to the structural complexity of activated sludge flocs, adapted DNA extraction protocols are often required for microbial composition analysis (Bourrain et al. 1999). Since Ca. *N. nitrosa* and Ca. *N. inopinata* form aggregates within these flocs, DNA extraction can be challenging (Kits et al. 2017). This study tested different protocols to enhance floc disintegration and DNA recovery. Sonication and agitation significantly improved extraction efficiency, though extending agitation time did not yield additional benefits. Filtering samples through a 0.45 μm pore-size membrane further concentrated DNA, increasing yield. PCR amplification of the COMAMMOX *amoA* gene region was successful for both May and November 2021 samples. The presence of Ca. *N. nitrosa* remained stable over time, consistent with findings by Cotto et al. (2023), who suggested wastewater-adapted strains. Ca. *N. inopinata* was detected only in November 2021 and in May 2021 samples subjected to intensive pre-treatment (20 min agitation, 15 min sonication, and filtration), indicating its localization within the inner floc regions, as hypothesized by Gao et al. (2018). Ca. *N. nitrificans* was identified solely in the May 2021 sample, possibly reflecting its temperature sensitivity (Wang et al. 2021). Additionally, the *amoA* gene clade A was detected in both samples, while clade B appeared exclusively in November 2021. The presence of COMAMMOX *Nitrospira* clade B was unexpected, given its usual association with forest ecosystems (Wang et al. 2019). These results underscore the necessity of optimized DNA extraction for reliable COMAMMOX detection via PCR.

To differentiate canonical NOB from *Nitrospira*, PCR assays targeted the *cynS* and *nxrB* genes. The *cynS* gene encodes cyanase, catalyzing cyanate conversion into ammonium and carbon dioxide. Although some COMAMMOX *Nitrospira* species harbor *cynS* (Spasov et al. 2020), its absence in most strains makes it a valuable marker for canonical NOB (Palatinszky et al. 2015; Jiang et al. 2020). Conversely, the *nxrB* gene encodes the NXR β-subunit and has historically been used as a biomarker for NOB. However, because *nxrB* sequences of strict COMAMMOX and NOB bacteria cluster within *Nitrospira* sublineage II, this marker cannot reliably distinguish between them (Jiang et al. 2020). The primer pair used in this study (*nxrB*169f/*nxrB*638r) specifically amplifies all *Nitrospira* lineages (Pester et al. 2014). Additionally, bacterial detection was enhanced using 16S rRNA gene primers (341F/785R) targeting hypervariable V3–V4 regions.

Quantification via digital PCR (dPCR) provided precise gene copy numbers. This technique, based on sample partitioning and fluorescence detection, enables absolute quantification through Poisson statistics (Hindson et al. 2013; Kanagal-Shamanna 2016). Results showed that the 16S rRNA gene had the highest copy number, followed by (1) *cynS* (NOB), (2) *nxrB* (*Nitrospira*), (3) *amoA* (AOB), (4) *amoA* (COMAMMOX clade A), (5) *amoA* (COMAMMOX clade B), and (6) *amoA* (AOA). The comparable abundance of canonical NOB and COMAMMOX genes suggests their coexistence under low ammonium conditions (Fujitani et al. 2020). The high *nxrB* copy number, associated with both COMAMMOX and NOB, may overestimate their relative abundance due to *Nitrospira* possessing 1–5 copies per genome (Jiang et al. 2020). Clade-specific variations in *amoA* copy numbers further reflected their distinct environmental adaptations (Palomo et al. 2018). AOA abundance was minimal (0.002%) but within expected ranges (Xu et al. 2021). These findings align with metataxonomic analysis and previous studies. Yao & Peng (2017) reported nitrifiers comprising 1–10% of total bacterial populations, with AOB averaging 1.27% and NOB 4.02%. In this study, nitrifiers accounted for 4.37% (January 2020: 1.22% AOB, 3.15% NOB) to 0.85% (November 2021: 0.17% AOB, 0.68% NOB). Observed biases likely stem from bioinformatics processing and incomplete COMAMMOX reference databases. Moreover, this analysis identified denitrifying bacteria, essential for nitrogen removal, with abundances ranging from 8.37% to 4.44%, reinforcing their role in nitrate reduction and overall nitrogen cycling.

In summary, in this research we used different techniques to gain a comprehensive understanding of COMAMMOX bacteria populations (and other nitrifiers) found in a WWTP bioreactor operating under conditions favorable for their development. Our study highlights that, despite the availability of molecular targets for monitoring COMAMMOX bacteria, a combination of different approaches and tools is necessary to obtain a complete profile of the nitrifying community, particularly for COMAMMOX bacteria. At present, FISH targets for COMAMMOX can only reliably detect *Ca. N. nitrosa* and *Ca. N. nitrificans* species. The results from PCR analysis depend on the primer sequences targeting *amoA*, which allow for the detection of COMAMMOX bacteria at various levels: population, clades (A or B), or species. Effective DNA extraction protocols are essential to prevent the loss of low-abundance or less accessible bacterial groups within the sample, ensuring reliable results. On the other side, the digital PCR technology was shown as the best approach to accurately detect and quantify the number of copies of the target genes selected for this study, revealing very low copies numbers, such as those for COMAMMOX clade B and AOA. Lastly, Illumina sequencing provides the complete taxonomic profile of the whole microbial community in the bioreactor, although its accuracy at the species level was limited, and COMAMMOX seem to be underestimated with this technique. However, one limitation of this study is that all techniques were not applied to every sample, making some results not entirely comparable across methods. Future research should include more frequent sampling and a standardized application of techniques to better understand the temporal dynamics of COMAMMOX bacteria and their interactions with other microbial groups. These findings highlight the necessity of integrating multiple techniques for accurate nitrifying community monitoring, ultimately aiding in the optimization of WWTP management.

## Electronic supplementary material

Below is the link to the electronic supplementary material.

Supplementary Material 1.

## Data Availability

The 16S rRNA amplicon sequencing raw data have been deposited in the National Center for Biotechnology Information (NCBI) under accession number PRJNA1232652.
