# Supplementary material for: Monitoring complete ammonia oxidizers bacteria: relevant players for nitrogen removal from wastewater
Source: AMB Express. 2025 Jul 4;15:101. doi: 10.1186/s13568-025-01878-6 (PMC12227399; doi:10.1186/s13568-025-01878-6)
Supplement: Supplementary file 1 — Supplementary Material 1. [file 13568_2025_1878_MOESM1_ESM.docx]

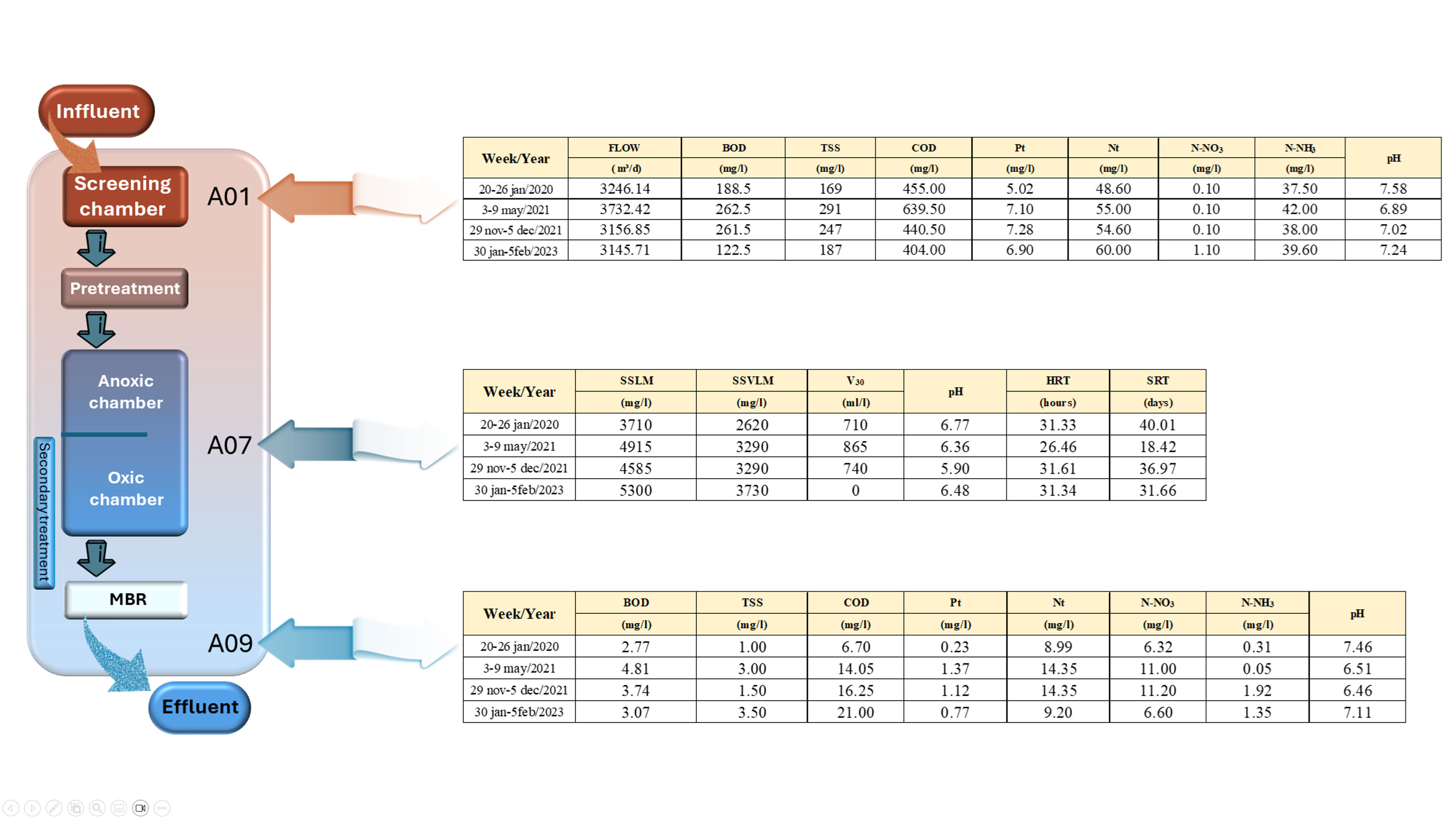


**Fig. S1. Schematic illustration of the full scale-WWTP.** Values recorded from the physical-chemical analysis of influent (A01), effluent (A09), and mixed liquor from the bioreactor oxic chamber (A07). Results for Hydraulic Retention Time (HRT) and Sludge Retention Time (SRT) are also included.


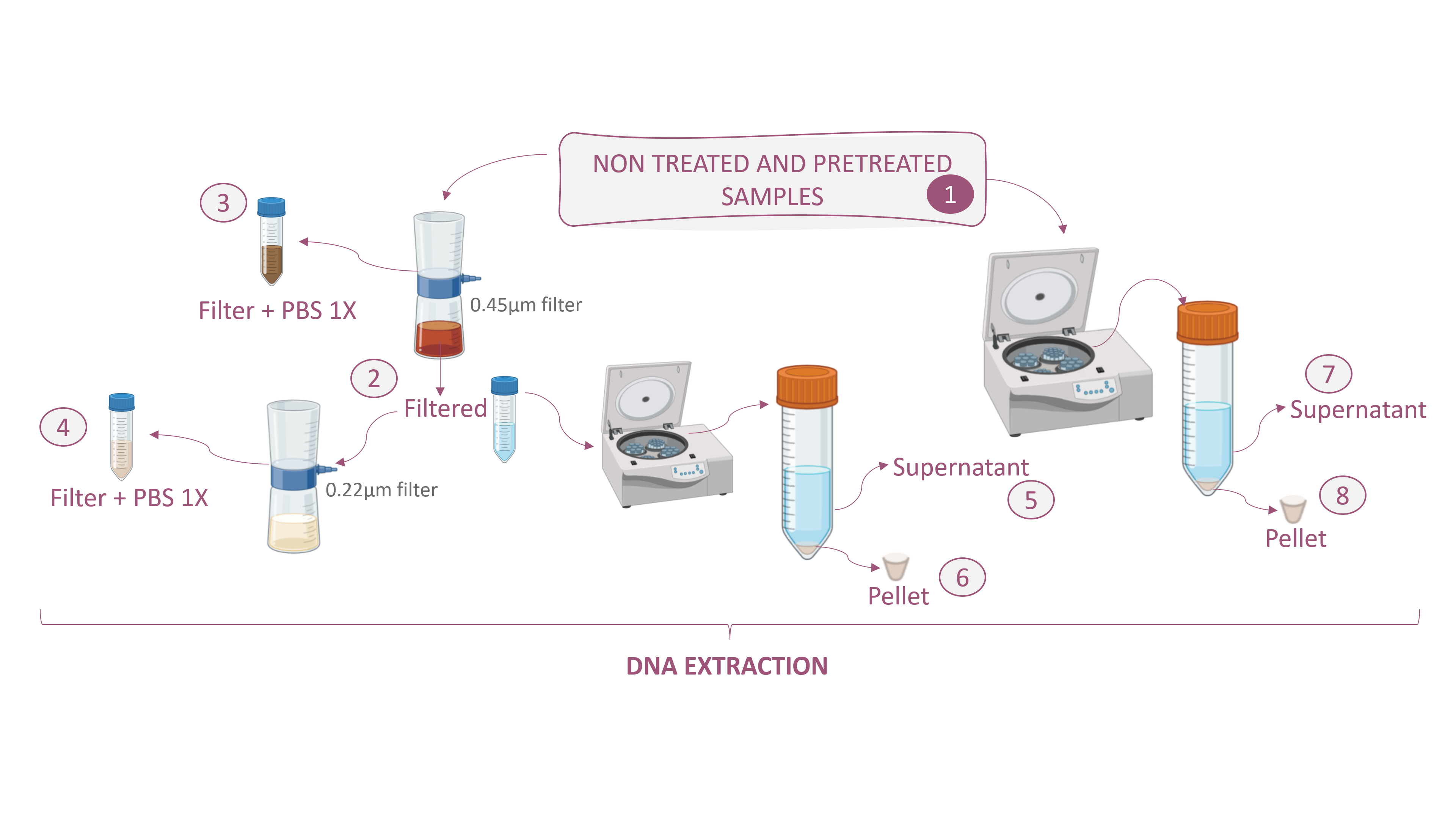


**Fig. S2.** Overview of alternative DNA extraction protocols applied to untreated and pretreated samples (MC, MA, and MB), collectively referred to as subsample 1. This initial sample was processed in parallel though two distinct workflows: filtration and centrifugation. In the filtration workflow, subsample 1 was first passed through a 0.45 µm membrane filter, generating two fractions: the filtrate (subsample 2) and the filter-retained fraction, which was resuspended in 1X PBS (subsample 3). Subsample 2 was subsequently subjected to a second filtration using a 0.22 µm membrane, producing a retained fraction that was also resuspended in 1X PBS (subsample 4). In parallel, subsample 2 was centrifuged, yielding a supernatant (subsample 5) and a pellet resuspended in saline solution (4% NaCl) (subsample 6). In the centrifugation workflow, subsample 1 was directly centrifuged, resulting in a supernatant (subsample 7) and a pellet, which was likewise resuspended in 4% NaCl saline solution (subsample 8). Each of these subsamples (indicated by circled numbers in the diagram) was processed in duplicate for DNA extraction.

**Table S1. FISH probes description (Greuter et al., 2016).** EUB338 probes were used in an equimolar mixture to detect all Bacteria. The percentage of formamide employed for optimal hybridization is also shown.

| **Bacteria** | **Probe** | ***E. coli* 16S rRNA gene position** | **Sequence (5´-3´)** | **Specificity** | **FA (%)** | **Reference** |
| --- | --- | --- | --- | --- | --- | --- |
| Eubacteria | EUB338 | 338-355 | GCT GCC TCC CGT AGG AGT | Domain *Bacteria* | 0-50 | Amann et al. (1990) |
|  | EUB338 II | 338-355 | GCA GCC ACC CGT AGG TGT | Order *Planctomycetales* | 0-50 | Daims et al. (1999) |
|  | EUB338 III | 338-355 | GCT GCC ACC CGT AGG TGT | Order *Verrucomicrobiales* | 0-50 | Daims et al. (1999) |
|  | EUB338 IV |  | GCA GCC TCC CGT AGG AGT | Bacterial lineages not covered by probes EUB338, EUB338II, and EUBIII | 0-50 | Schmid et al. (2005) |
| COMAMMOX | Ntspa476 | 476-494 | CTG CAG GTA CCG TCC GAA | *Ca. N. nitrosa, Ca. N. nitrificans* | 20 | Van Kessel et al. (2015) |
|  | CNtspa476 | 476-494 | CTG GAG GTA CCG TCC GAA | Competitor to Ntspa476 |  | Van Kessel et al. (2015) |
| NOB | Ntspa662 | 662-679 | GGA ATT CCG CGC TCC TCT | Genus *Nitrospira* | 35 | Daims et al.  (2001) |
|  | CNtspa662 | 662-679 | GGA ATT CCG CTC TCC TCT | Competitor to Ntspa662 |  | Daims et al.  (2001) |
|  | NIT3 | 1035-1052 | CCT GTG CTC CAT GCT CCG | Genus *Nitrobacter* | 40 | Wagner et al. (1996) |
|  | CNIT3 | 1035-1052 | CCT GTG CTC CAG GCT CCG | Competitor to NIT3 |  | Wagner et al. (1996) |
|  | Ntoga122 | 122–140 | TCCGGGTACGTTCCGATAT | Genus *Nitrotoga* | 40 | Lücker et al. (2014) |
|  | C1Ntoga122 | 122–140 | TCWGGGTACGTTCCGATAT | Competitor 1 to Ntoga122 |  | Lücker et al. (2014) |
|  | C2Ntoga122 | 122–140 | TCYGGGTACGTTCCGATGT | Competitor 2 to Ntoga122 |  | Lücker et al. (2014) |
| AOB | Nso1225 | 1224-1243 | CGC CAT TGT ATT ACG TGT GA | Betaproteobacterial ammonia-oxidizing bacteria | 45 | Mobarry et al. (1996) |
|  | Nmv (Ncmob) | 174-191 | TCC TCA GAG ACT ACG CGG | *Nitrosococcus mobilis* (*Nitrosomonas*) lineage | 35 | Juretschko et al. (1998) |
|  | Nsv443 | 444-462 | CCG TGA CCG TTT CGT TCC G | *Nitrosospira* spp. | 30 | Mobarry et al. (1996) |
|  | NEU | 653-670 | CCC CTC TGC TGC ACT CTA | Most halophilic and halotolerant *Nitrosomonas* spp. | 40 | Wallner et al. (1993) |
|  | CNEU | 653-670 | TTC CAT CCC CCT CTG CCG | Competitor to NEU |  | Wallner et al. (1993) |

**Table S2. Primers used for the detection of Total COMAMMOX, *Ca. N. nitrosa*, *Ca. N. inopinata* and *Ca. N. nitrificans*, COMAMMOX clades A and B, key nitrifying genes (*amoA*, *cynS*, *nxr*), and universal 16S rRNA bacteria gene.** The amplified gene and the sequence of each of them (direct and reverse) are indicated. Degenerate bases: B, C/G/T; Y, C/T; R, A/G; N, A/T/C/G; S, C/G; H, A/C/T; M, A/C; K, G/T; W, A/T. In grey, targets amplified using the ddPCR technique.

| **Gene Target** | **Primer Name** | **Primer Sequence (5’-3’)** | **Primer Sequence (3’-5’)** | **Reference** |
| --- | --- | --- | --- | --- |
| *amoA* gene of COMAMMOX (total COMAMMOX) | Ntsp-amoA 162F/359R | GGATTTCTGGNTSGATTGGA | WAGTTNGACCACCASTACCA | (Fowler *et al*., 2017) |
| *amoA* gene of *Ca. Nitrospira nitrosa* | Nitrosa amoA- 469F/812R | GCGATTCTGTTTTATCCCAGCAAC | CCGTGTGCTAACGTGGCG | (Keene-Beach *et al*. 2019) |
| *amoA* gene of *Ca. Nitrospira inopinata* | Nitrosa amoA- 410F/815R | TCACCTTGTTGCTAACTAGAAACTGG | TCCGCGTGAGCCAATGT |  |
| amoA gene of *Ca. Nitrospira nitrificans* | Nitrosa amoA- 463F/836R | ATGTTCGCGGCACTGTT | CCAGAAAGTTTAGCTTTGTCGCCT |  |
| amoA gene of Comammox *Nitrospira* clade A | CA377f- C576r | GTGGTGGTGGTCBAAYTA | GAAGCCCATRTARTCNGCC | (Jiang et al., 2020) |
| *amoA* gene *of* Comammox *Nitrospira* clade B | CB377f- C576r | GTACTGGTGGGCBAAYTT | GAAGCCCATRTARTCNGCC | (Jiang et al., 2020) |
| *cynS* gene of sNOB | Ntspa-cynSF- Ntspa-cynSR | TSATCGGHGTSTAYGGMGA | CCGTTCARSGTRATCTTGCA | (Jiang et al., 2020) |
| *amoA* gen *of* AOA | Arch-amoAF- Arch-amoAR | STAATGGTCTGGCTTAGACG | GCGGCCATCCATCTGTATGT | (Francis et al., 2005) |
| amoA gen of AOB in Betaproteobacteria | amoA1F- amoA2R | GGGGTTTCTACTGGTGGT | CCCCTCKGSAAAGCCTTCTTC | (Rotthauwe et al., 1997) |
| nxr gen of *Nitrospira* | nxrB169F- nxrB638R | TACATGTGGTGGAACA | CGGTTCTGGTCRATCA | (Pester et al., 2014) |
| Bacteria RNA 16S gene | 341F-785R | CCTACGGGNGGCWGCAG | GACTACHVGGGTATCTAATCC | (Klindworth et al., 2013) |
